# Maternal depressive symptoms, attendance of sessions and reduction of home safety problems in a randomized toddler safety promotion intervention trial: A latent class analysis

**DOI:** 10.1371/journal.pone.0261934

**Published:** 2022-01-19

**Authors:** Yan Wang, Eric Zhu, Erin R. Hager, Maureen M. Black

**Affiliations:** 1 Department of Pediatrics, University of Maryland School of Medicine, Baltimore, Maryland, United States of America; 2 Department of Epidemiology and Public Health, University of Maryland School of Medicine, Baltimore, Maryland, United States of America; 3 Centennial High School, Ellicott City, Maryland, United States of America; 4 RTI International, Research Triangle Park, North Carolina, United States of America; Erasmus Medical Center, NETHERLANDS

## Abstract

**Objective:**

Little is known about the association between maternal depressive symptoms and attendance at safety promotion interventions. This study used latent class analysis (LCA) to identify the profile of attendance within a toddler safety intervention and assessed its relation with maternal depressive symptoms at baseline and reduction of home safety problems over time, separately.

**Methods:**

The analytic sample included 91 mothers of toddlers (mean maternal age 28.16 years) who were assigned to the safety promotion intervention group as part of a randomized trial and assessed at baseline, 6-month and 12-month follow-ups. Using LCA, we classified mothers into low and high attendance classes based on their attendance at 8 intervention sessions. We assessed maternal depressive symptoms with the Beck Depression Inventory (BDI) and home safety problems with a 9-item home safety problem observation.

**Results:**

The mothers were classified into low attendance (45%) and high attendance classes (55%). The posterior probability of attending each session ranged from 0–0.29 for the low attendance class and 0.68–0.92 for the high attendance class. Each one unit increase of BDI sum score at baseline was associated with an 8% reduced odds of being in the high attendance class (aOR = 0.92, 95% CI: 0.86, 1.00, p = 0.037). The home safety problem score reduction was greater among high attendance class participants than low attendance class participants at the 6-month follow-up (b = -1.15, 95% CI:-2.09, -0.20, p = 0.018).

**Conclusion:**

Maternal depressive symptoms were associated with the reduced probability of maternal attendance at toddler safety promotion sessions; high session attendance was related to greater reduction of toddler home safety problems. Identifying risk factors for maternal low attendance to interventions and developing strategies to promote attendance should lead to reductions in home safety problems and reductions in unintentional injuries among young children.

## Introduction

Unintentional injuries are the number one cause of mortality among children 1–19 years-of-age in the U.S. [1]. The burden is especially high for young children 1–4 years-of-age [1]. Home safety problems (e.g, lack of smoke alarm or unsafe storage of poisonous substances) are associated with a greater risk of unintentional injuries among young children, indicating that reducing home safety problems can reduce injuries among young children [2]. Although multiple safety promotion interventions have been conducted to prevent unintentional injuries among young children (e.g., distribution of home safety devices and parental education on home safety), a systematic review reported inconsistent effects [3]. Low parental attendance to the interventions may contribute to the inconsistent findings in child safety promotion interventions, consistent with findings in broader behavior intervention research that low attendance reduces intervention effectiveness [4].

Understanding factors related to parent attendance within safety prevention interventions is a critical step in maximizing intervention impacts and reducing unintentional injuries. Depressive symptoms have been related to low adherence to lifestyle interventions and treatment among adults or adolescents with overweight/obesity [5–7] and may be linked to low attendance in toddler safety promotion interventions. Mothers of young children, especially those in low-income households, are vulnerable to depressive symptoms [8]. However, little research has examined whether depressive symptoms predict maternal attendance to toddler safety promotion interventions.

In a previous paper, we reported that mothers of toddlers from low-income households randomized to a safety promotion intervention had a significantly greater reduction of home safety problems compared to an attention control group [9]. However, we did not assess the factors related to attendance of the safety promotion intervention and whether attendance was related to the reduction of home safety problems. This paper extends our previous research by examining maternal depressive symptoms and other socio-demographic factors in relation to maternal intervention attendance. We also assessed mothers’ attendance in relation to the reduction of home safety problems over time. We used latent class analyses (LCA) to classify the participants into attendance classes, given the lack of valid cutoff on high versus low attendance. We hypothesized that (1) high levels of maternal depressive symptoms predict lower intervention attendance, compared to low levels of maternal depressive symptoms and (2) high intervention attendance is related to greater reduction in home safety problems over time, compared to low intervention attendance.

## Materials and methods

We retrieved data from a randomized controlled trial to promote toddler safety [9, 10], which was registered at ClinicalTrials.gov as “Toddler Overweight Prevention Study Among Low-Income Families” with the identifier number as NCT02615158. In brief, a sample of 277 biological mothers of toddlers was recruited in 2007–2010 from a Special Supplemental Nutrition Program for Women, Infants and Children clinic serving a semiurban community and a pediatric primary care clinic serving a low-income urban community. After a cohort of ~30 dyads completed baseline, we used a randomization procedure to assign them to the safety promotion intervention group or the two obesity prevention groups that served as attention-control. The intervention was initiated shortly after recruitment of each cohort and conducted over 3–4 months. We recruited 9 cohorts, enrolling 91 into the safety promotion intervention and 186 into the attention-control groups. For the total sample in the trial (n = 277), mothers’ mean age was 27.3 years and the toddler’s mean age was 20.1 months. 81% of the mothers completed high school/equivalent and 69% lived at/below the federal poverty line. The consort flow diagram, eligibility of the participants, priori sample size calculation for the original trial, detailed description of the trial design including the randomization procedure and data collection procedures are reported previously [9].

The current study used a subsample of the mother-toddler dyads who were assigned to the safety promotion intervention group (n = 91). The protocol for the current study can be found at protocols.io (dx.doi.org/10.17504/protocols.io.bmsek6be). The study was approved by the ethical review board of University of Maryland, Baltimore and the ethical review board of Maryland Department of Health. All mothers provided written consent.

We conducted the toddler safety promotion intervention at two community sites and focused on four toddler safety areas: fire prevention, fall prevention, poison control and car seat use [9]. Following the Triple P- Positive Parenting Program [11], a highly effective evidence-based parenting programs, the intervention included eight sessions led by health educators grounded in social cognitive theory principles, which emphasizes interactions among people (personal factors), their behavior, and their environments [12]: five in-person group sessions including a final review/celebration session, and three individual phone sessions. Group activities were designed to help mothers build safety knowledge, encourage peer modelling, promote perceived importance in toddler safety, provide social support and build self-confidence in adopting safety strategies. During each session, participants established goals to improve toddler safety. After four in-person group sessions, health educators conducted individual phone sessions to discuss the participants’ safety goals and work with them individually to develop strategies to achieve their goals. The final group session was a review of all the previous topics and a celebration.

### Measures

#### Attendance

Health educators recorded the participants’ attendance at each of the five in-person group sessions and each of the three phone calls (1 = yes or 0 = no).

#### Maternal depressive symptoms

Maternal depressive symptoms were measured at baseline with the Beck Depression Inventory (BDI) [13]. The BDI includes 21 questions relating to depressive symptoms in the past week, e.g., sadness and feeling guilty, using a 4- point Likert scale ranging from 0 to 3. We summed the scores to calculate a sum score, with higher scores indicating more symptoms.

#### Home safety problems

During home visits, data collectors (research assistants) observed home safety problems at baseline, before the intervention, and at 6- and 12-months after baseline with high inter-rater reliability (>90%). Nine items were observed with the score as 1 = yes/0 = no: (1) exposed wires; (2) covered outlets; (3) working smoke detector on each floor; (4) stair gate for toddlers in homes with stairs; (5) dangerous balcony/porch; (6) unsafe outside handrails/steps/stairs; (7) chipped/peeled paint; (8) peeling paint/broken plaster >81/2 by 11 inches and (9) child-resistant latches on cleaning supply/medication cabinets [9]. Several items (item 2, 3, 4, 9) were reverse coded. The data collectors asked participants if they could observe the home and some participants refused inspection of certain areas of the homes. Over three fourth of the homes were observed without missing values for any items at baseline, first and second follow-ups, respectively [9]. Missing items were substituted with participant-level mean. There is no recommendation for stair gate use for toddlers ≥24 months and the score for stair gate was also substituted with participant-level mean if there was no stair at home or the toddler was over age 24 months at the home observation. A summary score of the nine items was calculated, ranging from 0 to 9. It was treated as a continuous variable in the analyses with a higher score indicating more safety problems.

#### Socio-demographic variables

Mothers self-reported their age, education, and marital status, and their toddler’s sex, race/ethnicity, and birth date. We calculated the toddler’s age based on mother-reported birth date and the interview date. The poverty threshold is provided by the US Census Bureau as the total household income, given a specific family size, and a number of related children under 18 years [14]. Mothers reported total household income, family size and number of children in the household. The Federal poverty ratio was estimated by calculating the ratio of the total household income for each mother-toddler dyad versus the total household income at the poverty threshold, given the same family size, and the number of related children under 18 years. Urban/semi urban residence was based on recruitment site [9].

### Analyses

We conducted descriptive analyses, including frequencies, means and standard deviations for the attendance, home safety problems, and depressive symptoms. We used LCA with Mplus 8.0 statistical software to estimate the heterogeneity (latent classes) in the attendance of 8 sessions among the participants by separating the sample into conditionally independent latent classes [15, 16]. There were no missing values in attendance. We used LCA to classify participants into latent classes based on their attendance, rather than using a continuous variable to indicate the number of sessions attended for three reasons. First, a continuous variable gives each session equal weight and does not consider that some sessions have low attendance (e.g. less attendance on phone call sessions than in-person group sessions). Second, a continuous variable does not consider measurement error in each session. Third, the participants’ attendance was not normally distributed and there was no existing arbitrary cutoff (e.g. any attendance vs. no attendance) in relation to the significant safety promotion intervention effect. We estimated the number of classes based on 8 variables indicating attendance to each of the 8 sessions, and did not include any predictors of the class membership in the models. Due to a modest sample size, we compared 1-class, 2-classes, 3-classes LCA. The best model was selected based on the following criteria: (1) the likelihood-based statistics, e.g. lower Akaike information criterion [AIC] or Bayesian information criterion [BIC], (2) Vuong-Lo-Mendell-Rubin (VLMR) Likelihood Ratio Test (LRT) and Bootstrapped Likelihood Ratio Test (BLRT), which compared a model with K classes to the model with K-1 classes, with a non-significant result indicating that the model with K-1 classes is preferred as it does not fit the data worse than the model with K classes, and (3) the percentage of the smallest class (greater than 5%) [15, 16]. We used a Chi-square statistic to test the null hypothesis that the model does fit the data well for each model.

After deciding the LCA with the optimal number of classes, we conducted a 3-step approach LCA with auxiliary variables (predictors of latent class membership, including sociodemographic variables, baseline depressive symptoms and baseline safety problems). The latent class membership did not change after including the auxiliary variables [17]. However, inclusion of these auxiliary variables can be used to assess the relations between these variables and the latent class membership. In the 3-step approach, the latent class model is estimated in a 1^st^ step with only latent class indicator variables; in the 2^nd^ step, the most likely class variable is created using the latent class posterior distribution obtained during the 1^st^ step; in the 3^rd^ step, the most likely class is regressed on predictors based on the multinomial logistic regression, accounting for the misclassification in the 2^nd^ step [18]. All the three steps can be implemented automatically by specifying “AUXILIARY = x(R3STEP)” command, where the AUXILIARY option specified variables that are not part of the analysis, but were important predictors of the latent classes using the three-step approach and the R3STEP indicated that the specified x variables will be used as covariates (predictors) in the third step of the logistic regression (for 2 classes) or multinomial logistic regression (for > = 3 classes) in the 3-step LCA [15]. Compared to the traditional one-step approach, the three-step approach accounts for classification errors to prevent underestimation of the association between covariates and class membership [18].

Finally, we output the latent class membership and assessed the change of home safety problem score over time between each pair of classes based on linear mixed modeling (LMM) with STATA 16.0 statistical software. The LMM included the home safety problem score at the three assessments as the outcome, and time (3-category), latent class membership and the interaction between time and latent class membership as predictors, accounting for the clustering of the repeated measures within each participant by specifying a random intercept for each participant. Maximum Likelihood Estimation (MLE) accounted for missingness in the continuous outcome variables to provide unbiased parameter estimates [19]. An alpha of 0.05 was used as the cutoff for statistical significance.

The sample size estimation for LCA depends on how well the classes are separated. In statistical power analyses, enough power is usually defined as 1- β with a minimum of 0.8, where β indicates the probability of a type II error. A simulation study suggested that a sample size of ~100 provides enough power (> = 0.8) to differentiate the classes if there are medium to large effects (Cohen’s w > = 0.44) in a 2-class LCA model and large effects (Cohen’s w > = 0.84) in a 3-class LCA [20]. In addition, a post-hoc power analysis suggested that the sample size of 91 provides enough power to detect a medium association between attendance class and depressive symptoms or change in home safety problems for either a 2 or 3-class model [21].

## Results

The characteristics of the sample in this study (n = 91) are shown in Table 1. The mean age of the mothers was 28.16 (SD = 5.99) years, with 58% residing in urban and 42% in semi-urban communities. The majority (82%) graduated from high school or completed a GED (Graduate Education Degree test). The mean poverty ratio was 0.88 and 32% of the mothers were married. Cronbach’s alpha was 0.91 on the BDI, indicating good internal consistency. The average score for the BDI was 8.57 (SD = 7.13) and for home safety problems was 2.36 (SD = 1.58). The mean age for the toddlers was 20.14 months (SD = 5.46), divided evenly by sex, and 68% were Black. A total of 65/91 (71%) mothers attended ≥1 session. The number (percent) of attendance by number of sessions was: 1 session (n = 5 participants, 5%), 2 sessions (n = 7, 8%), 3 sessions (n = 4, 4%), 4 sessions (n = 3, 3%), 5 sessions (n = 7, 8%), 6 sessions (n = 8, 9%), 7 sessions (n = 15, 16%), and all 8 sessions (n = 16, 18%); 29% of the participants did not attend any sessions.

**Table 1:** Selected sample characteristics in relation to latent class membership (n = 91).

	Total(n = 91)	Low attendance class(n = 41)	High attendance class(n = 50)	aOR	95% CI	p
***Maternal characteristics***						
Age, mean (SD)	28.16(5.99)	27.86(6.62)	28.41(5.49)	1.02	0.94–1.10	0.712
Residence n(%)						
Urban	53(58)	26(49)	27(51)			
Semi-urban	38(42)	15(39)	23(61)	1.45	0.47–4.44	0.518
Maternal education n(%)						
No high school diploma	16(18)	6(38)	10(63)			
High school diploma/equivalent or higher	75(82)	35(47)	40(53)	0.69	0.17–2.81	0.602
Poverty ratio, mean(SD)	0.88(0.77)	0.81(0.73)	0.93(0.81)	1.32	0.70–2.50	0.390
Marital status, n(%)						
Single, divorced, widowed	62(68)	29(47)	33(53)			
Married	29(32)	12(41)	17(59)	1.07	0.30–3.80	0.912
Baseline depressive symptoms, mean (SD)	8.57(7.13)	10.12(7.33)	7.30(6.77)	0.92	0.86–1.00	0.037
***Toddler characteristics***						
Age, mean (SD)	20.14(5.46)	20.45(5.34)	19.88(5.59)	0.99	0.91–1.08	0.826
Gender, n(%)						
Female	45(49)	22(49)	23(51)			
Male	46(51)	19(41)	27(59)	1.24	0.48–3.23	0.661
Race/ethnicity, n(%)						
White, Hispanic, or other	29(32)	13(45)	16(55)			
Non-Hispanic Black	62(68)	28(45)	34(55)	1.81	0.45–7.28	0.402
***Baseline home safety problem score*, *mean (SD)***	2.36(1.58)	2.43(1.52)	2.31(1.64)	1.07	0.80–1.45	0.641

Note: The percentages do not sum to 100% in row 10 due to rounding. aOR: adjusted Odds Ratio.

### Latent class membership

We assessed the model fit for the 1-class, 2-class, 3-class LCA, specifying the number of initial stage starts and final stage optimizations as 500 and 100, respectively. The best loglikelihood value has been replicated in each model. As shown in Table 2, the Chi-square test was significant for the 1-class model, but not for the 2-class or 3-class models. AIC was the smallest for the 3-class model and BIC was the smallest for the 2-class model. VLMR LRT and Bootstrapped LRT were both significant comparing the 3-class model to the 2-class model or the 2-class model to the 1-class model. Based on these criteria, both the 2-class and 3-class models were acceptable. However, after inspecting the 3-class model, the additional class included 4.4% (n = 4) of the participants who were divided from the high attendance class in the 2-class model, and had a high likelihood of attending all sessions, except the final celebration. Considering the small size of the 3^rd^ class, we chose the 2-class model as the final model. In the 2-class model, the entropy was 0.993, indicating good classification quality.

**Table 2:** Model fit criteria comparing 1-class, 2-class, 3-class latent class analysis model.

Models	Chi-square (df)	p	AIC	BIC	Sample-Size Adjusted BIC	% for the smallest class	Entropy	VLMR LRT	Bootstrapped LRT
**1-class**	2991.80(247)	<0.001	1007.33	1027.41	1002.16	---	---	---	---
**2-class**	266.96(236)	0.08	654.31	697.00	643.34	45%	0.99	<0.001	<0.001
**3-class**	169.77(228)	1.00	636.52	701.81	619.74	4%	1.00	0.023	<0.001

*AIC: Akaike information criterion. BIC: Bayesian information criterion, VLMR LRT: Vuong-Lo-Mendell-Rubin Likelihood Ratio Test. BLRT: Bootstrapped Likelihood Ratio Test.

**Fig 1:**
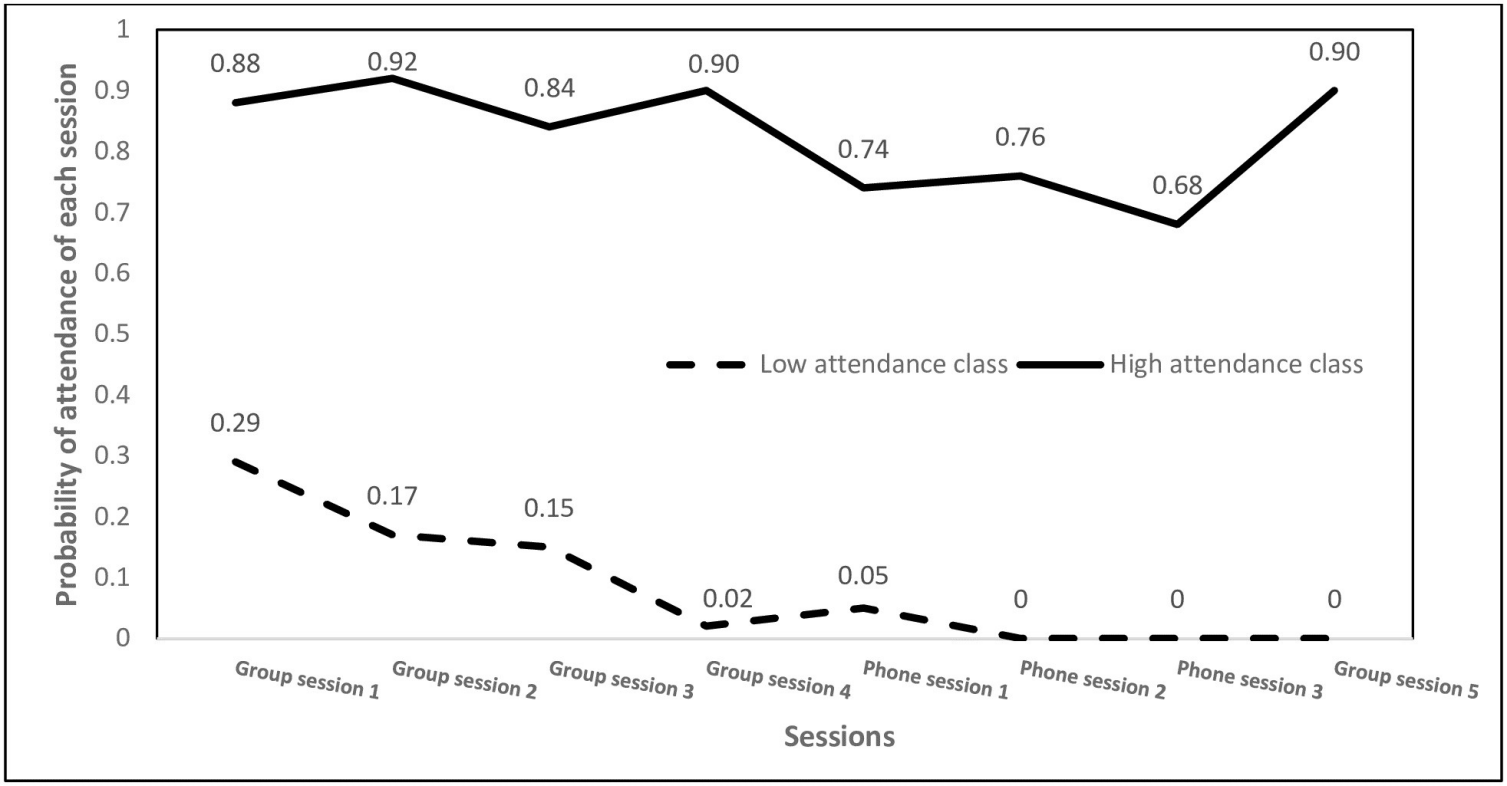
Probability of attendance for each session by latent class membership. Note: Group session 1 (introduction and car seat safety), group session 2 (poison hazard), group session 3 (fire prevention), group session 4 (fall prevention), group session 5 (review and celebration). The two lines indicate the observed probabilities of attendance of each session for “low attendance class” and “high attendance class”, separately.

We included auxiliary variables in the 2-class LCM. There were two classes of attendance, indicating a low attendance class (n = 41, 45%) and a high attendance class (n = 50, 55%). As shown in Fig 1, in the low attendance class, the probability of mothers attending the group sessions varied from 0–0.29 and was highest for the 1^st^ session (0.29) and gradually decreased for each later session (0.17, 0.15, 0.02 and 0, separately). The proportion of mothers attending the individual phone sessions varied within 0–0.05. In the high attendance class, the proportion of mothers attending the group sessions varied between 0.84–0.90 (0.88, 0.92, 0.84, 0.90 and 0.90 separately for each session) and the proportion of mothers attending the individual phone sessions varied between 0.68–0.76. The mothers in the high attendance class attended a total of 3–8 sessions, with 2% (n = 1) within the class attending 3 sessions, 6% (n = 3) 4 sessions, 14% (n = 7) 5 sessions, 16% (n = 8) 6 sessions, 30% (n = 15) 7 sessions, and 32% (n = 16) 8 sessions. The mothers in the low attendance class attended a total of 0–3 sessions with 63% within the class (n = 26) attending no sessions, 12% (n = 5) 1 session, 17% (n = 7) 2 sessions, 7% (n = 3) 3 sessions.

### Latent class membership in relation to depressive symptoms

We assessed the relation between the latent class membership with depressive symptoms, initial home safety problem score and other covariates in the 3-step LCA. As shown in Table 1, the only significant predictor of class membership is the depressive symptom score. In the low attendance class, the mean score of initial depressive symptoms was 10.12 (SD = 7.33); in the high attendance class, the mean score of initial depressive symptoms was 7.30 (SD = 6.77). With one unit increase of initial depressive symptoms, there was 8% reduced likelihood of being in the high attendance class (adjusted Odds Ratio, aOR = 0.92, 95% CI: 0.86–1.00, p = 0.037), after controlling for other predictors in Table 1. The difference in mean score of initial depressive symptoms between the two classes was estimated as 0.4 times of the standard deviation of the initial depressive symptoms (Cohen’s d~ = 0.40, medium-small effect size [22]). The initial home safety problem score was not related to the latent class membership (mean score 2.43 vs. 2.31, aOR = 1.07, 95% CI: 0.80–1.45, p = 0.641). Other maternal or toddler characteristics were not related to the latent class membership.

### Latent class membership in relation to home safety problem score at the follow-ups

**Fig 2:**
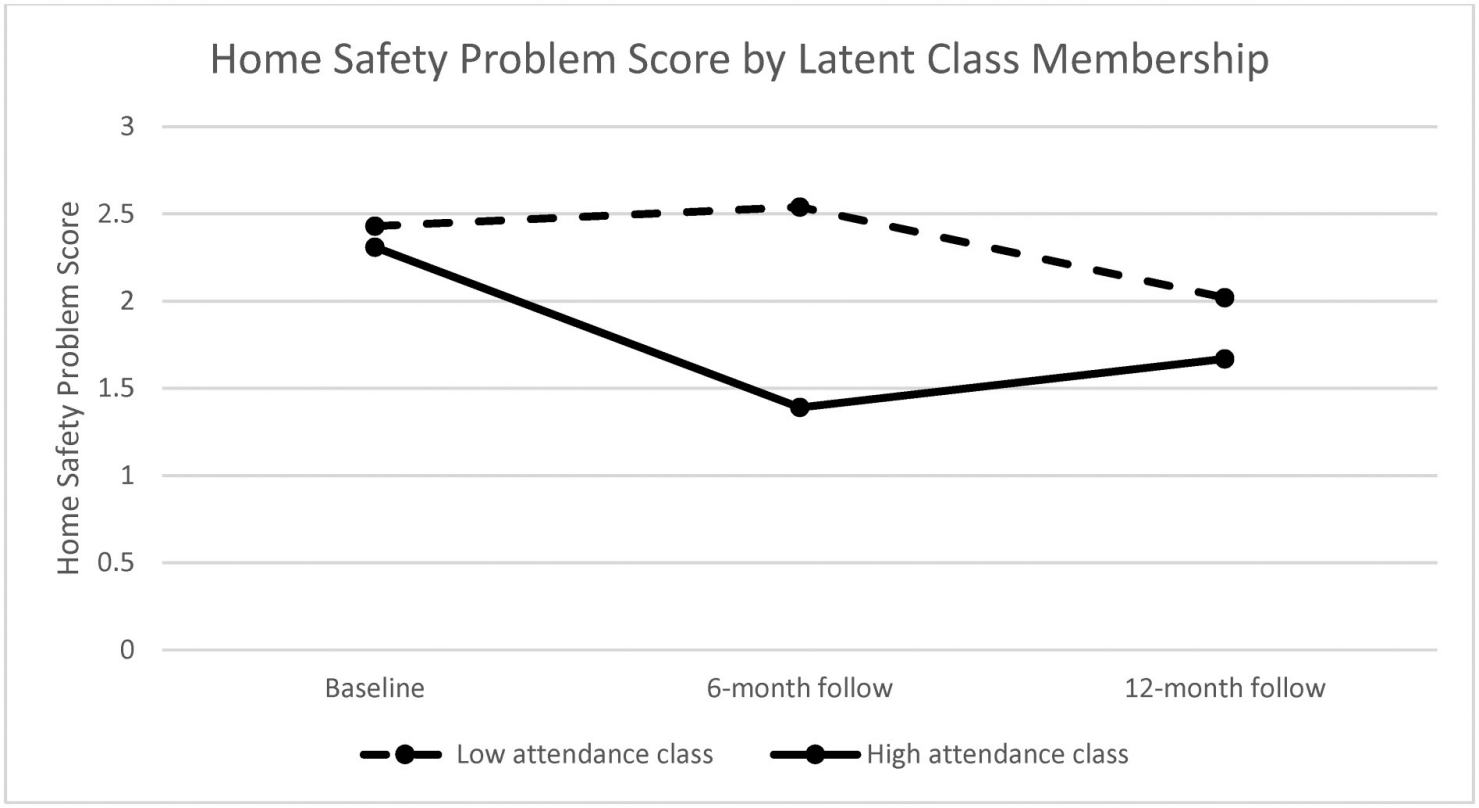
Home safety problem score at each assessment by latent class membership.

As shown in Table 3, the mean (SD) of the safety problem score was 2.43 (SE = 0.17), 2.54 (SE = 0.55) and 2.02 (SE = 0.28) at baseline, 6-month and 12-month follow, separately, for the participants in the low attendance class, and 2.31 (SE = 0.23), 1.39 (SE = 0.17) and 1.67 (SE = 0.22), separately, for the participants in the high attendance class. Fig 2 lists the home safety problem scores at the three assessments by the latent classes. No significant difference was found between the two classes in the home safety problem score at baseline. The home safety problem score was higher in the low attendance class than the high attendance class at the 6-month follow (p = 0.011), not at the 12-month follow (p = 0.319) based on independent T-tests.

**Table 3:** Linear mixed model on home safety problem score in relation to latent class membership.

Home safety problem score	Total	Low attendance class	Change over time in low attendance class*	p	High attendance class	Change over time in high attendance class*	p	Between-class difference in change over time	p
	Mean (SE)	Mean (SE)	Mean (95% CI)		Mean (SE)	Mean (95% CI)		Mean (95% CI)	
Baseline	2.36(0.17)	2.43(0.24)			2.31(0.23)				
6-month follow	1.73(0.21)	2.54(0.55)	0.26(-0.51, 1.04)	0.502	1.39(0.17)	-0.88(-1.43, -0.34)	0.002	-1.15(-2.09, -0.20)	0.018
12-month follow	1.81(0.17)	2.02(0.28)	-0.40(-1.03, 0.23)	0.215	1.67(0.22)	-0.68(-1.21, -0.15)	0.012	-0.28(-1.10, 0.54)	0.506

Note:

*The change is from baseline to 6-month follow up and baseline to 12-month follow up, separately.

As shown in Table 3, the linear mixed regression showed that there was not a significant change in the home safety problem score from baseline to either follow-up within the low attendance class. In contrast, the home safety problem score decreased significantly from baseline to 6-month follow-up (b = -0.88, 95% CI: -1.43, -0.34, p = 0.002) or 12-month follow-up (b = -0.68, 95% CI: -1.21, -0.15, p = 0.012) within the high attendance class. The high attendance class had significantly more reduction in home safety problems compared to the low attendance class at the 6-month follow-up (b = -1.15, 95% CI: -2.09, -0.20, p = 0.018). The difference in change between the two classes was not significant at the 12-month follow-up (p>0.10, Table 3, Fig 2).

## Discussion

This study has three main findings: (1) two patterns of maternal attendance (high and low attendance classes) were identified in the toddler safety promotion intervention, (2) maternal depressive symptoms predicted low attendance class membership, and (3) high attendance class was related to greater reduction of home safety problems at the 6-month, but not at the 12-month follow-up, compared to the low attendance class.

First, using LCA, we identified two unique patterns of maternal attendance in the toddler safety promotion intervention (high vs. low attendance classes). LCA has been used to assess adherence patterns for other behaviors, e.g. adherence to diet and exercise recommendations in adults with overweight or obesity [23] and adherence to antipsychotic drug therapy in patients [24]. Few studies have used LCA to assess intervention attendance. The traditional analytic approach to attendance depends on the number of sessions attended, treating all sessions in a similar manner with no differentiation, and arbitrarily defining a cutoff for the number of sessions [7]. LCA is a person-centered, model-based approach. In this paper, we used LCA to classify the mothers based on their attendance to each unique session, and organized mothers with similar attendance patterns into two classes. In this study, LCA indicates that attendance of at least 4 out of 8 sessions (about 50% attendance) increases the probability of resulting in an intervention effect on the reduction of home safety problems. Future studies could use a similar approach to understand the link between attendance and other health behavior changes.

Second, mothers with higher depressive symptoms were more likely to be in the low attendance class, regardless of their initial score on the measure of home safety problems. This finding is consistent with several other studies that reported depressive symptoms in relation to intervention attendance [5–7]. Although the mechanism underlying the association between depressive symptoms and attendance is unknown, there are several possibilities. In addition to physical symptoms associated with depression (e.g. fatigue and pain) [25], symptoms of sadness, loss of interest in daily activities, and difficulty thinking clearly may interfere with responsive parenting [26], including attendance at a safety promotion intervention. Other explanations cannot be excluded. Common factors such as low socioeconomic status increases maternal stress which could lead to depressive symptoms and low behavioral intervention attendance [8, 27]. However, we controlled for socio-demographic variables, illustrating that the relation of maternal depressive symptoms to attendance was independent of socio-demographic variables.

Third, the high attendance class had significantly lower home safety problem scores and experienced significantly greater reduction in home safety problems at the 6-month follow-up compared to the low attendance class. At baseline home safety scores did not differ between the two classes. The reduction in scores among the high attendance class suggests that attendance was responsible for the improvement at the 6-month follow-up. At the 12-month follow-up, the improvement had faded, which is consistent with other studies that reported a fadeout in the intervention effect over time for interventions targeting behavioral improvements [28]. Among the low attendance class, the changes at the 6-month and 12-month follow-ups were not significant, suggesting that attendance was necessary for improvement and that depressive symptoms interfered with attendance.

This study has several strengths: First, it fills a gap in research by examining the risk factors associated with maternal attendance within a toddler safety intervention. Second, the study addressed a sample of mother-toddler dyads from low-income households, an understudied group at high risk of unintentional injuries. Third, attendance was recorded by health educators at each session, which reduced potential recall bias by the participants. Fourth, a variety of potential confounders, including maternal age, education, and socio-demographic variables were included. Fifth, instead of using an arbitrary cutoff for attendance, we used a statistical method LCA to classify participants into groups based on the attendance patterns.

The limitations of this study need to be noted. First, the sample size does not allow us to examine additional classes, which might provide more refined information on attendance beyond the low and high attendance classes. Second, we did not assess home rental status. The participants in the sample were mainly Black and from low-income households. Black households have a higher rate of house rentership than white households [29]. Rentership has been related to higher risk of pediatric injuries [30, 31]. In addition, housing discrimination and bias can also affect the quality of housing for Black households by limiting their access to housing [32]. Power structures, including those by landlords, may limit the ability of Black households to modify their home safety environment [33–35]. Future studies should account for rental status in assessing home safety problems. Third, the findings were based on a sample of mothers of toddlers from low-income households, who are at risk of both high depressive symptoms and home safety problems. The findings need to be replicated before being generalized to mothers of toddlers from other socio-economic groups.

## Clinical implications

Little is known about the factors related to maternal attendance of interventions to promote toddler safety. This study contributes to the understanding that maternal depressive symptoms relate to attendance, and in turn, attendance relates to the intervention effect. Mothers of young children from low-income households are at increased risk for depressive symptoms [8]. Effective strategies need to be adopted to reduce depressive symptoms and to promote attendance of safety promotion interventions for preventing unintentional injuries among young children. Future studies may assess the underlying mechanisms linking maternal depressive symptoms to the low attendance of intervention sessions among mothers from low-income households and assess maternal attendance in toddler safety promotion intervention among mothers from different sociodemographic backgrounds.

## Supporting information

S1 Checklist CONSORT 2010 checklist of information to include when reporting a randomised trial. (DOC)

S1 File CONSORT diagram (this is the same consort diagram in a paper we published in 2018 to assess the overall safety promotion intervention effect). (DOCX)
