# Supplementary material for: Maternal depressive symptoms, attendance of sessions and reduction of home safety problems in a randomized toddler safety promotion intervention trial: A latent class analysis
Source: PLoS One. 2022 Jan 19;17(1):e0261934. doi: 10.1371/journal.pone.0261934 (PMC8769292; doi:10.1371/journal.pone.0261934)
Supplement: S1 File — (DOCX) [file pone.0261934.s002.docx]

**Consort Diagram for the randomized toddler safety promotion intervention trial**

**(The current study used a subsample of mother-toddler dyads who were assigned to the safety intervention (n=91)).**

Flow of participants who were recruited, randomly assigned, and followed for safety assessment in a safety promotion prevention program among a sample of mother-toddler dyads from low-income families (n=277).

Randomized (N=277)

(including 8 mother-dyads who refused home safety observation)

Randomized to safety intervention (N=91)

Attended >= 1 safety intervention session (n=65, 71%)*

Did not attend any intervention sessions (n=26, 29%)

Randomized to attention control (obesity intervention, N=186)

Attended>=1 allocated obesity intervention session (n=131, 70%)

Did not attend any obesity intervention sessions (n=55, 30%)

Lost to 6-month follow-up

(n=37, 41%)

Lost to 6-month follow-up (n=59, 32%)

Lost to 12-month follow-up

(n=21, 23%)

Lost to 12-month follow-up

(n=42, 23%)

Assessed for eligibility

(N=509)

Excluded (N=232)

Ineligible (n=4)

Active/passive refusal (n=201)

Did not complete baseline

assessment (n=27).

Followed-up at 6 months

(n=54, 59%)

Followed-up at 12 months

(n=70, 77%)

Followed-up at 6 months

(n=127, 68%)

Followed-up at 12 months (n=144, 77%)

Note: This is cited from a published paper: Wang Y, Gielen AC, Magder LS, Hager ER, Black MM. A randomised safety promotion intervention trial among low-income families with toddlers. Injury Prevention. 2018;24(1):41-7.
